# Stress Distribution in 5-Unit Fixed Partial Dentures with a Pier Abutment and Rigid and Nonrigid Connectors with Two Different Occlusal Schemes: A Three-Dimensional Finite Element Analysis

**DOI:** 10.1155/2023/3347197

**Published:** 2023-02-01

**Authors:** Behnaz Ebadian, Amirhossein Fathi, Shivasadat Tabatabaei

**Affiliations:** ^1^Dental Implants Research Center, Department of Prosthodontics, School of Dentistry, Isfahan University of Medical Sciences, Isfahan, Iran; ^2^Department of Prosthodontics, Dental Materials Research Center, Dental Research Institute, School of Dentistry, Isfahan University of Medical Sciences, Isfahan, Iran; ^3^Dental Students' Research Committee, School of Dentistry, Isfahan University of Medical Sciences, Isfahan, Iran

## Abstract

**Objectives:**

This study aimed to assess stress distribution in 5-unit fixed partial dentures (FPDs) with a pier abutment and rigid (RC) and nonrigid connectors (NRCs) with the canine rise and group function occlusal schemes by finite element analysis (FEA).

**Materials and Methods:**

In this FEA study, a geometrical model of the maxilla with natural teeth and periodontal ligament (PDL) was three-dimensionally designed and meshed by ANSYS and Pro/Engineer software programs. A 5-unit FPD was then designed to replace the lost first premolar and first molar teeth; the second premolar served as a pier abutment, and the canine and second molar served as terminal abutments. Two FPDs were designed with RC and NRC. Each FPD was analyzed with the canine rise and group function occlusal schemes (a total of 4 models). The first and second molars (180 N), premolars (120 N), and canine (80 N) teeth were subjected to progressive vertical and oblique (12-degree) loads, and maximum von Mises stress and strain in teeth and connectors were calculated for each model.

**Results:**

The models had 73704 elements and 137732 nodes. The connector design and occlusal scheme had significant effects on stress distribution in FPDs. The highest von Mises stress (73.035 MPa) was recorded in FPD with RC and group function occlusal scheme. The lowest von Mises stress (0.004 MPa) was recorded in FPD with NRC and canine rise occlusal scheme.

**Conclusion:**

Oblique forces created greater stress, and FPD with NRC and canine rise occlusal scheme decreased stress in FPD and increased stress in the tooth crown.

## 1. Introduction

Considering the high prevalence of partial and complete edentulism in adults, replacement of the lost teeth is a common task for dental clinicians [1]. Some partially edentulous patients require the replacement of the lost teeth with fixed partial dentures (FPDs) with a pier abutment [2]. A Pier abutment is a natural tooth located between two edentulous spaces and terminal abutments, which supports an FPD. For instance, the second premolar can serve as a pier abutment when the first premolar and first molar teeth are lost [3]. Obviously, the optimal function of FPDs depends on balanced stress distribution in the pier abutment and terminal abutments.

Rigid connectors (RCs) are commonly used for FPDs [1]. When occlusal loads are applied to the connectors, the pier abutment can serve as a support, and result in debonding of the terminal abutment with lower retention, leading to restoration fracture and treatment failure [2]. Thus, FPDs with RCs may not be an appropriate treatment option for the replacement of two lost teeth with one pier abutment, because The amount of load applied to the anterior part may be different from the posterior part, causing the destruction of the prosthesis, tooth decay, pulp irritation, and ultimately the need for root canal treatment, causing prolonged pain and discomfort to the patient [4–12]. Also, failure may occur due to differences in the physiological mobility of the teeth in the anterior and posterior regions [13]. Thus, nonrigid connectors (NRCs) have been proposed to decrease the risk of failure [2, 14]. NRCs transfer stress to the supporting bone instead of its concentration in the connector [15]. However, NRCs have drawbacks as well, such as greater bone loss around the pier abutment, higher cost, and requiring a longer time for fabrication [16].

Occlusion is a highly debated topic in the fabrication of FPDs. In lateral movements of the mandible, the mandibular posterior teeth come out of centric occlusion with the opposing teeth. This movement is guided by the condyle in the posterior region and follows a lateral downward path in the anterior region as dictated by the anterior guidance [17, 18]. The dental occlusal scheme is conventionally divided into four groups anterior guidance, canine rise, partial group function, and group function. In the canine rise occlusal scheme, only the maxillary and mandibular canine teeth are in contact in lateral excursive movements while in group function, only the posterior teeth are allowed to be in contact during lateral movements, and at least canine and two premolars at the working side should be in contact with the opposing teeth [19]. Sidana [17] and other supporters of the group function occlusal scheme believe that occlusal wear in this scheme is a compensatory coping mechanism that results in optimal stress distribution for an appropriate functional relationship [17].

Clinical assessment of stress concentrated in prosthetic components or transferred to the supporting tissue is almost impossible. However, since the success of prosthetic restorations is influenced by biomechanical properties, measurement of these stresses is critical [2, 20]. Photo-elastic models are important tools for the assessment of stress distribution in different systems. Nonetheless, these models are made of plastic material, and cannot precisely simulate the biological behavior of tissues. Thus, the pattern of stress distribution in the enamel, dentin, and pulp, and the nonlinear behavior of the periodontal ligament cannot be simulated. Finite element analysis (FEA) provides new insight into tooth biomechanics, and can three-dimensionally simulate the behavior of many dental structures with different shapes and properties under different loading conditions [2]. Fathi et al. [2] evaluated stress distribution in a 5-unit FPD with a pier abutment under constant and progressive loads. They designed FPDs with RC and NRC and applied vertical and oblique loads to them. They showed different patterns of stress distribution under vertical and oblique loads and revealed that NRC decreased stress in restoration and increased stress in crestal bone. Oruc et al. [21] used FEA to assess stress distribution in a 5-unit FPD with pier abutment and RC and NRC. The second molar and canine teeth served as terminal abutments, and the second premolar served as a pier abutment with a mesial and distal connector. They showed that NRC at the distal of the second premolar resulted in less stress concentration following the application of 50 N static load to the cusps. Jiang et al. [22] assessed the durability of two resin-bonded FPDs with RC and NRC by the wear test. The first premolar and second molar teeth served as abutments for 4-unit FPDs. NRC was placed at the distal of the first premolar. The models underwent thermal cycles and mechanical loading. The results showed that NRC decreased the stress between tooth and wing since it allowed independent movement of the two separate prosthetic components, and increased the durability and clinical service of FPD. Although some articles suggest the application of new materials for force distribution in the dental prosthesis, changes in the design of the prosthesis can also be important in this distribution [23].

Implant-tooth-supported fixed partial prostheses can cause changes in marginal bone loss under the effect of changes of occlusal forces [24] and the available studies on stress distribution in 5 unit FPDs with a pier abutment and RC and NRC did not address the role of different occlusal schemes in stress distribution. In addition, the finite element articles on this subject exclusively examine the restoration of the mandible, but the direction of the occlusal forces in the maxilla and mandible is different [25]. Thus, this study aimed to assess stress distribution in maxillary 5-unit FPDs with a pier abutment and RC and NRC with the canine rise and group function occlusal schemes by FEA.

## 2. Materials and Methods

In this FEA study, a geometrical model of the maxilla was designed by ANSYS (ANSYS Inc.; Houston, TX, USA) and Pro/Engineer (Parametric Technology Corporation, USA) software programs. Approval was obtained from the Isfahan University of Medical Sciences ethics committee (IR.MUI.RESEARCH.REC.1400.344).

### 2.1. Modeling in Mimics and 3Matic Software Programs

The bone, teeth, metal framework, and connectors were modeled in Mimics and 3Matic software programs. For this purpose, cone-beam computed tomography scans with 1 mm slice interval were imported to Mimics. A 3D model of the maxillary canine, first and second premolar, and first and second molar is simulated based on the information obtained from Wheelers's dental anatomy, physiology, and occlusion [26, 27]. External shapes of the roots were generated from the textbook [27]. A cement-retained Straumann CARES titanium abutment with a gingival height of 1.5 mm and a Straumann bone level tapered (BLT) implant with a diameter of 4.1 mm and a height of 10 mm were designed and positioned in the maxillary canine site. The implant was presumed to be completely osseointegrated. The tooth was prepped conventionally for a PFM restoration. The crowns were composed of Ni-Cr alloy and porcelain and zinc phosphate cement used for both the tooth and implant. The segmentation tool was used to create masks for the teeth, maxilla, and PDL, and then Calculate 3D feature was used to design a 3D model of the components. All components were then exported in STL format and converted to STP format in Geomagic software by reverse engineering.

### 2.2. Analysis in ANSYS Software

After the conversion of all geometries to STP format, they were imported to ANSYS software.

The designed geometrical model had natural teeth, PDL, and maxillary bone three-dimensionally. The model was then meshed in the software and the mechanical properties of the materials were also uploaded for stress analysis. In FEM analysis, the stress distribution is commonly specified as von Mises stress, which could be maximum and minimum principal stress or principal strain. A formula is used to calculate the von Mises stress in three planes: the *x*-axis, the *y*-axis, and the *z*-axis. A 5-unit FPD was designed for the replacement of a lost first premolar and first molar. The second premolar served as a pier abutment, and the canine and second molar teeth served as terminal abutments. Two FPDs were simulated one with RC and the other one with NRC. To simulate NRCs, Shillingburg's dovetail design was used on the distal surface of the pier abutment. Next, each designed model was analyzed with two different occlusal schemes, namely canine rise and group function (a total of 4 models).

### 2.3. Boundary Conditions for Canine Rise Occlusal Scheme

To simulate the canine rise occlusal scheme, an 80 N load was applied to the canine tooth first vertically, and then with 12-degree angulation. The upper surface of the maxilla was fixed.

### 2.4. Boundary Conditions for Group Function Occlusal Scheme

Loads equal to 80 N, 120 N, 120 N, 180 N, and 180 N were applied to the canine, first premolar, second premolar, first molar, and second molar teeth, respectively, to simulate the group function occlusal scheme. The loads were first applied vertically, and then with 12-degree angulation. The upper surface of the maxilla was fixed [2].

### 2.5. Meshing

The total number of elements of the model was 73,704 tetrahedral elements, and the total number of nodes was 137,372 nodes. All materials were considered homogeneous, isotropic, and linearly elastic.

### 2.6. Sensitivity Analysis of Meshing

A sensitivity analysis curve was drawn to ensure the independence of results from the size of the meshing and the number of elements. For this purpose, the number of elements was increased (the size of elements decreased) and the von Mises stress value was read. The stress (MPa) curve was drawn based on the number of elements. The curve became flat when the number of elements reached 73,704. Thus, this value was considered as the optimal number of elements for meshing.

### 2.7. Properties of the Materials

Table 1 presents the mechanical properties of the materials used in this study according to the available literature [2, 28].

Next, maximum von Mises stress, maximum shear stress, maximum von Mises strain, and maximum shear strain around the teeth and connectors were calculated for the four models and compared.

## 3. Results

### 3.1. FPD with NRC and Canine Rise Occlusal Scheme (Figure 1)

Table 2 presents the von Mises stress and strain values in FPD with NRC and canine rise occlusal scheme.

### 3.2. FPD with NRC and Group Function Occlusal Scheme (Figure 2)

Table 3 presents the von Mises stress and strain values in FPD with NRC and group function occlusal scheme.

### 3.3. FPD with RC and Canine Rise Occlusal Scheme (Figure 3)

Table 4 presents the von Mises stress and strain values in FPD with RC and canine rise occlusal scheme.

### 3.4. FPD with RC and Group Function Occlusal Scheme (Figure 4)

Table 5 presents the von Mises stress and strain values in FPD with RC and group function occlusal scheme.

## 4. Discussion

According to Badwaik and Pakhan [26] each restoration should be able to withstand functional and parafunctional forces applied in the oral cavity. This factor should be taken into account in the fabrication of FPDs. If the loads applied to dental abutments exceed the physiological tolerance threshold of the supporting bone, they can cause bone loss and lead to treatment failure [29]. Thus, the treatment plans should include strategies to minimize such stresses. FEA can greatly help in this regard [30]. This study assessed stress distribution in 5-unit FPDs with a pier abutment and RC and NRC with the canine rise and group function occlusal schemes by FEA. The results showed that connector design had a significant effect on stress distribution in 5-unit FPD with pier abutment. The use of NRCs slightly decreased the level of stress in FPD at the site of connector attachment and cervical margin of restoration but increased the stress at the level of alveolar bone. According to Misch [31], in conventional FPDs, the male part of NRC is usually at the mesial surface of the posterior artificial tooth while the female part is located at the distal of the natural tooth abutment; this design prevents mesial movement of the connector [31].

With respect to stress and strain distribution in the abutments, the highest von Mises stress in the abutments (32.927 MPa) was noted on the buccal surface of the second premolar in both RC and NRC while minimum von Mises stress (0.0208 MPa) was recorded on the occlusal surface of second molar in FPD with NRC and canine rise occlusal scheme. Comparison of stress distribution in different designs showed high-stress concentration in the connectors and cervical part of the abutments, particularly the pier abutment. Stress concentration was recorded on root surfaces and the apical part. Nonetheless, NRC decreased the level of stress. This was specifically true when an NRC was placed at the distal of the pier abutment and decreased maximum stress concentration in the pier abutment. This design caused no stress accumulation in the anterior abutment with posterior loading, and vice versa. This finding may indicate the role of NRC in the prevention of the lever effect in a 5-unit FPD. Also, the stress level of the second molar was lower than that of the pier abutment and canine tooth, which may be explained by the larger periodontal area of the molar tooth compared with canine [32, 33] which may improve stress distribution as explained by Oruc et al. [21].

With respect to stress and strain distribution in FPDs, the highest von Mises stress value (63.959 MPa) was recorded on canine tooth crown in the use of NRC with group function occlusal scheme while the lowest von Mises stress (0.0426 MPa) was recorded on the occlusal surface of the second molar crown in use of NRC and canine rise occlusal scheme. In the present study, FPDs were subjected to progressive loads to simulate the mean masticatory forces applied in vivo. The present results were in agreement with those of Yoda et al. [34] who reported higher occlusal forces in the posterior region. According to their study, 180 N load was applied to molar teeth, 120 N to premolars, and 80 N to the canine tooth in the present study. This type of loading was practiced to more precisely simulate the masticatory forces applied to restorations in the clinical setting [20].

With respect to stress and strain distribution in the connectors, the maximum von Mises stress value (59.802 MPa) in NRCs was recorded in FPD with group function occlusion, and the minimum value (1.06 MPa) was noted in canine rise occlusion. The present results showed minimum stress in NRC with posterior loading condition, which was in line with previous findings [14, 25]. Ferencz [35] reported that resin-bonded FPDs with NRCs enabled independent movement of components, and were successful in the short term. Nonetheless, due to the placement of an NRC at the mesial surface of the abutment or distal surface of the anterior abutment, excessive stress concentration occurs in the anterior terminal abutment. A molar tooth has a larger periodontal surface than a canine [36] as explained earlier [37], and it may serve as an advantage for the molar tooth. Thus, stress concentration in the anterior abutment, compared with a posterior abutment, is less desirable. The present study showed stress concentration in canine with this particular location of NRC. Considering all the above, it may be stated with certainty that NRCs can decrease stress at the connectors and cervical part of FPDs but at the expense of increased stress in the crown, which subsequently increases the risk of bone loss [38–40]. The results of FEA are reported by the von Mises stress values, which indicate the entire stress in the field, and are considered as an index for possible trauma [25]. Since connectors are the site of maximum stress concentration in FPDs, NRCs are recommended [37, 41], and were simulated in the present study.

Concerning stress and strain distribution in bone, maximum von Mises stress in bone (73.035 MPa) was recorded in FPDs with RC and group function scheme while minimum value (0.004 MPa) was recorded in FPDs with NRC and canine rise scheme. Yoda et al. [34] performed photoelastic analysis on the bone with NRCs and reported that stress concentration points varied depending on the location of NRCs. The present results were in line with their findings. They also added that FPDs with RCs distributed the stresses homogenously and vertically, and NRCs located at the distal surface of the canine and mesial surface of molar teeth caused uniform stress distribution. Consistent with their findings, the present results revealed more uniform stress distribution in the loading of all teeth in FPDs with RC. Stress distribution in the use of NRCs located at the distal surface of the canine and mesial surface of the molar tooth was similar to the use of RC and group function loading. RC and NRC have differences in stress distribution and concentration in the supporting bone [34, 37, 40]. Stress distribution in bone in the present study was similar to previous findings [34, 37, 40]. FPDs with RC and pier abutments can serve as a lever, and create a high-stress concentration in pier abutments, which can result in excessive movements in terminal abutments and their subsequent damage. Thus, NRCs can be used to eliminate the role of pier abutments as support [37]. Ideally, an NRC should be located at the distal surface of the pier abutment [14, 25].

The occlusal scheme is the most important factor which should be taken into account prior to treatment to maximize the possibility of a favorable prognosis [8, 28]. According to the present results, NRC with a canine rise occlusal scheme would be the optimal design for a 5-unit FPD with a pier abutment.

This study had some limitations. Due to its nature, all components were considered homogeneous, isotropic, and linearly elastic [33], which is not the case in the clinical setting. Also, the results were compared qualitatively. In total, the inherent limitations of FEA should be considered when interpreting the results. Also, due to the *in vitro* design of the study, the results cannot be directly generalized to the clinical setting. Future studies on additional loading conditions on the occlusal surface and connectors or research on more demanding crown materials including zirconia crowns and the effect of these forces on this type of crown material are needed, considering the properties of zirconia and its challenging adhesion to the teeth [42]. Moreover, the most common location of NRC on the abutment was simulated in this study [14]. Future studies may focus on other designs of NRCs and their effect on stress distribution.

## 5. Conclusion

The NRC generated less stress than RC in this study. Oblique forces created greater stress, and maximum stress was noted in the canine abutment. Deformation was greater in pier abutments (compared with terminal abutments), and in RC (compared with NRC). Stress was greater in pier abutment and NRC, compared with RC. The FPD with NRC and canine rise occlusal scheme decreased stress in FPD and increased stress in the tooth crown, and can be suggested as the best design for a 5-unit FPD with pier abutment.

## Figures and Tables

**Figure 1:**
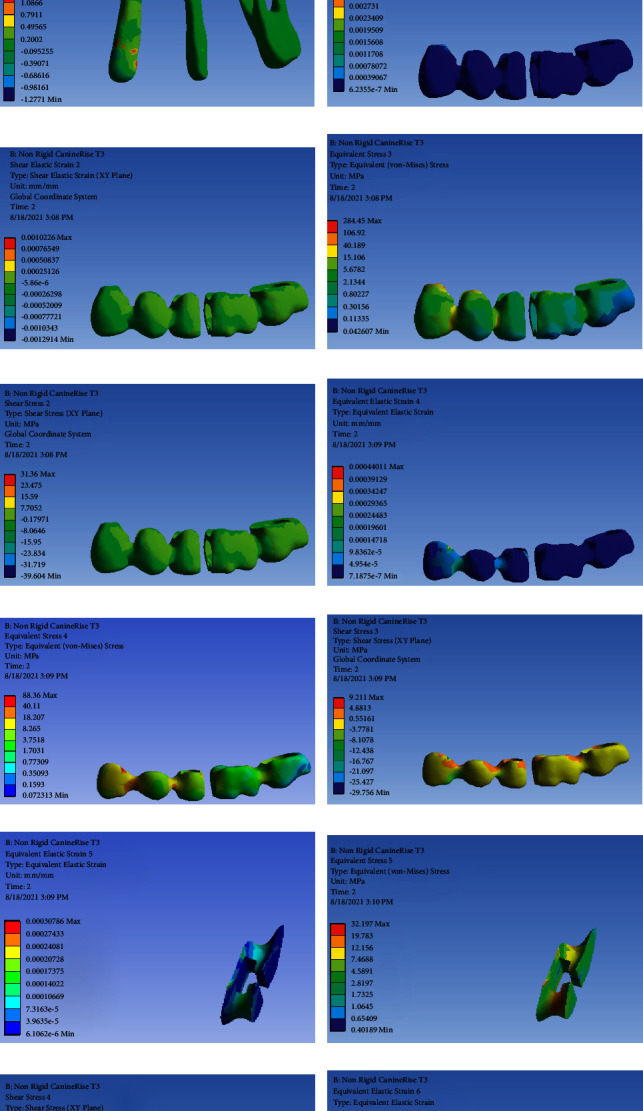
FPD with NRC and canine rise occlusal scheme: (a) strain distribution in the entire model, (b) von Mises stress distribution in the entire model, (c) strain distribution in abutments, (d) von Mises stress distribution in abutments, (e) shear stress distribution in abutments, (f) strain distribution in FPD, (g) strain distribution in FPD, (h) von Mises stress distribution in FPD, (i) shear stress distribution in FPD, (j) strain distribution in the metal framework, (k) von Mises stress distribution in the metal framework, (l) shear stress distribution in the metal framework (m) strain distribution in the connector, (n) von Mises stress distribution in the connector, (o) shear stress in the connector, (p) strain distribution in the connector, (q) von Mises stress distribution in the connector, and (r) shear stress distribution in the connector.

**Figure 2:**
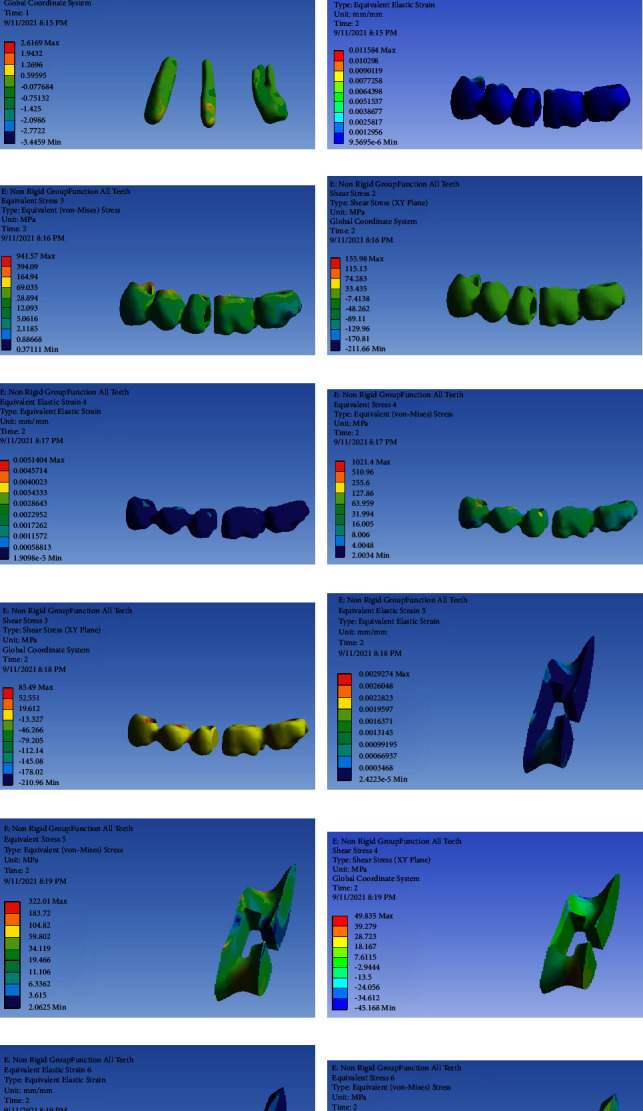
FPD with NRC and group function occlusal scheme: (a) strain distribution in the entire model, (b) von Mises stress distribution in the entire model, (c) strain distribution in abutments, (d) von Mises stress distribution in abutments, (e) shear stress distribution in abutments, (f) strain distribution in FPD, (g) von Mises stress distribution in FPD, (h) shear stress distribution in FPD, (i) strain distribution in the metal framework, (j) von Mises stress distribution in the metal framework, (k) shear stress distribution in the metal framework, (l) strain distribution in the connector, (m) von Mises stress distribution in the connector, (n) shear stress distribution in the connector, (o) strain distribution in the connector, (p) von Mises stress distribution in the connector, and (q) shear stress distribution in the connector.

**Figure 3:**
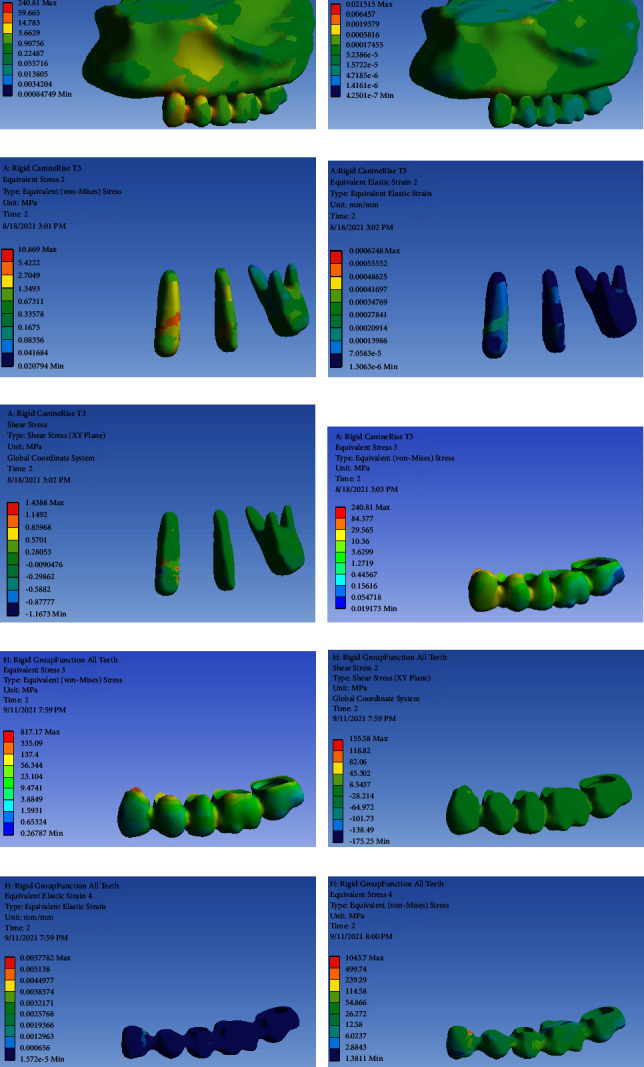
FPD with RC and canine rise occlusal scheme: (a) von Mises stress distribution in the entire model, (b) strain distribution in the entire model, (c) von Mises stress distribution in abutments; (d) strain distribution in abutments, (e) shear stress distribution in abutments, (f) von Mises stress distribution in FPD, (g) strain distribution in FPD and shear stress distribution abutments, (h) strain distribution in the metal framework, (i) von Mises stress distribution in metal framework, and (j) shear stress distribution in the metal framework.

**Figure 4:**
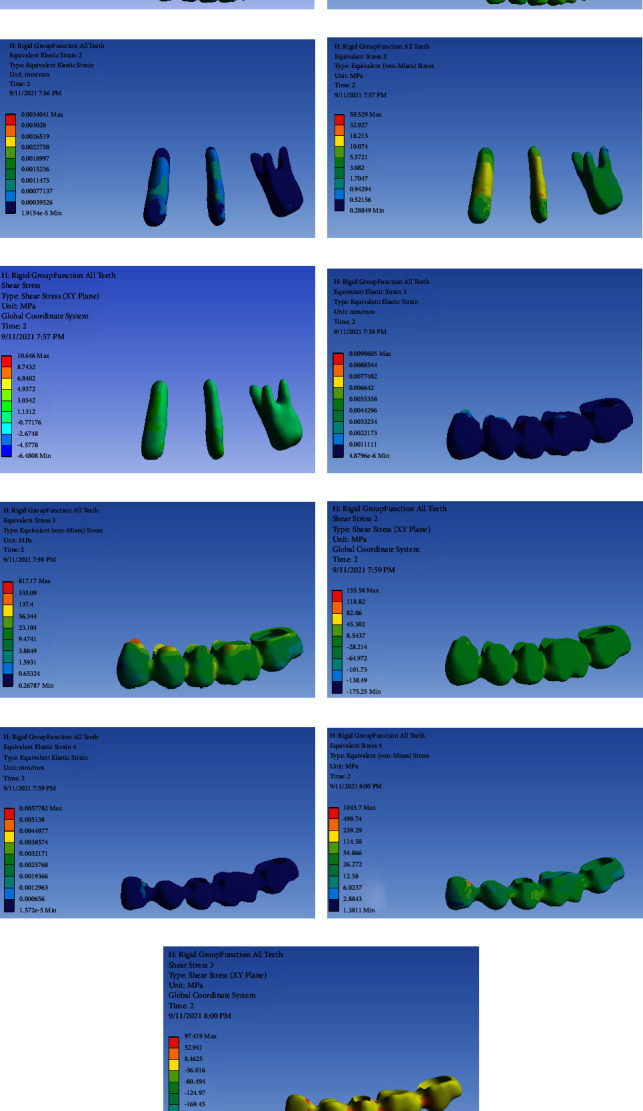
FPD with RC and group function occlusal scheme: (a) strain distribution in the entire model, (b) von Mises stress distribution in the entire model, (c) strain distribution in abutments, (d) von Mises stress distribution in abutments, (e) shear stress in abutments, (f) strain distribution in FPD, (g) von Mises stress distribution in FPD, (h) shear stress distribution in FPD, (i) strain distribution in the metal framework, (j) von Mises stress distribution in the metal framework, and (k) shear stress distribution in the metal framework.

**Table 1:** Mechanical properties of the materials used in this study.

Component	Young's modulus (MPa)	Poisson's ratio
Cortical bone	13700	0.3
Cancellous bone	1370	0.3
Cobalt-chromium metal framework	206000	0.33
Porcelain	82800	0.35
PDL	69	0.45
Tooth	18000	0.33
Nonrigid connector	110000	0.33

**Table 2:** Von Mises stress (MPa) and strain values in FPD with NRC and canine rise occlusal scheme.

Variable	Component	Maximum value	Location of maximum value	Minimum value	Location of minimum value
Stress	Entire model	17.657	The mesial surface of the first premolar and the distal surface of the canine	0.004	The occlusal surface of the second molar
	Tooth	5.3259	The buccal surface of the canine	0.0208	The occlusal surface of the second molar
	FPD	40.189	The mesial surface of the first premolar and the distal surface of the canine	0.0426	The occlusal surface of the second molar
	Metal framework	40.11	The mesial surface of the first premolar and the distal surface of the canine	0.1593	The occlusal surface of the second molar
	Connector	15.216	The mesial surface of the connector	1.06	The distal superior surface of the connector

Strain	Entire model	0.006	The distal surface of the canine and mesial surface of the first premolar	1.991	The occlusal surface of the second molar
	Tooth	0.0002	The lingual surface of the canine	1.529	The occlusal surface of the second molar
	FPD	0.007	The lingual surface of the canine	6.235	The occlusal surface of the first and second molars
	Metal framework	0.0003	The distal surface of the second premolar	7.187	The occlusal surface of the first and second molars
	Connector	0.0002	The buccal inferior surface of the connector	3.963	The lingual superior surface of the connector

**Table 3:** Von Mises stress (MPa) and strain values in FPD with NRC and group function occlusal scheme.

Variable	Component	Maximum value	Location of maximum value	Minimum value	Location of minimum value
Stress	Entire model	67.888	The distal surface of the second premolar and the mesial surface of the first molar	0.077	The occlusal surface of the second molar
	Tooth	32.501	The mesial surface of canine and second premolar	0.525	The distal root of the second molar
	FPD	69.035	The buccal surface of the canine	0.886	The distal surface of the second molar
	Metal framework	63.959	The distal surface of the canine	8.006	The buccal surface of the second molar
	Connector	59.802	Mesial surface	2.0625	Superior, distal and buccal surface of the connector

Strain	Entire model	0.020	The buccal surface of the second premolar	4.462	The occlusal surface of the entire FPD
	Tooth	0.001	The buccal surface of the canine and second premolar	1.924	The occlusal surface of the second molar
	FPD	0.003	The distal surface of the canine	9.569	The occlusal surface of the entire FPD
	Metal framework	0.001	The distal surface of the canine	1.909	The occlusal surface of the entire framework
	Connector	0.001	The buccal surface of the connector	2.4223	The distal surface of the connector

**Table 4:** Von Mises stress and strain values in FPD with RC and canine rise occlusal scheme.

Variable	Component	Maximum value	Location of maximum value	Minimum value	Location of minimum value
Stress	Entire model	59.665	The distal surface of the canine and mesial surface of the first premolar	0.003	The occlusal surface of the second premolar
	Tooth	5.422	The buccal surface of the canine	0.083	The lingual root of the second molar
	FPD	29.565	The buccal surface of the canine	0.054	The occlusal surface of first and second molars
	Metal framework	38.361	The distal surface of the canine	0.045	The distal surface of the second molar

Strain	Entire model	0.0005	The distal surface of canine and first premolar	4.718	The buccal surface of the first and second molars
	Tooth	0.0002	The buccal surface of the canine	7.058	The terminal surface of all roots
	FPD	0.0006	Mesial surface of the canine	4.250	Occlusal surface of entire FPD
	Metal framework	0.0002	The distal surface of the canine and mesial surface of the first premolar	5.321	The occlusal surface of first and second molars

**Table 5:** Von Mises stress and strain values in FPD with RC and group function occlusal scheme.

Variable	Component	Maximum value	Location of maximum value	Minimum value	Location of minimum value
Stress	Entire model	73.035	The buccal surface of the first premolar and first molar	1.352	The occlusal surface of the second molar
	Tooth	32.927	The buccal surface of the second premolar	0.288	The distal root of the second molar
	FPD	56.344	The lingual surface of the canine	0.653	The mesial surface of the canine and occlusal surface of the second molar
	Metal framework	114.58	The mesial surface of the first molar and the distal surface of the second premolar	2.884	The occlusal surface of the second molar

Strain	Entire model	0.002	The distal surface of the second premolar	1.2661*e* − 5	The mesial surface of the canine and occlusal surface of the second molar
	Tooth	0.001	The buccal surface of the canine	1.915	The occlusal surface of the second molar
	FPD	0.004	The lingual surface of the canine	0.001	The mesial surface of the second molar
	Metal framework	0.002	The distal surface of the canine	1.572	The occlusal surface of the second molar

## Data Availability

The data are available through a request from the corresponding author: Amirhossein Fathi, Email: amir_alty@yahoo.com.
