# Corrigendum to “Stress Distribution in 5-Unit Fixed Partial Dentures With a Pier Abutment and Rigid and Nonrigid Connectors With Two Different Occlusal Schemes: A Three-Dimensional Finite Element Analysis”

**DOI:** 10.1155/ijod/9767959

**Published:** 2025-07-25

B. Ebadian, A. Fathi, S. Tabatabaei, “Stress Distribution in 5-Unit Fixed Partial Dentures With a Pier Abutment and Rigid and Nonrigid Connectors With Two Different Occlusal Schemes: A Three-Dimensional Finite Element Analysis,” *International Journal of Dentistry* 2023, no. 1 (2023): 1–15, https://doi.org/10.1155/2023/3347197.

In the article, Reference 2 was incorrect. The correct Reference 2 is below:

R. Modi, S. Kohli, K. Rajeshwari, S. Bhatia “A Three-Dimension Finite Element Analysis to Evaluate the Stress Distribution in Tooth Supported 5-Unit Intermediate Abutment Prosthesis With Rigid and Nonrigid Connector,” *European Journal of Dentistry* 9, no. 2 (2015): 255−261, https://doi.org/10.4103/1305-7456.156847.

We apologize for this error.

